# Inflammation biomarker discovery in Parkinson’s disease and atypical parkinsonisms

**DOI:** 10.1186/s12883-020-1608-8

**Published:** 2020-01-17

**Authors:** Anna Santaella, H. Bea Kuiperij, Anouke van Rumund, Rianne A. J. Esselink, Alain J. van Gool, Bastiaan R. Bloem, Marcel M. Verbeek

**Affiliations:** 1 Departments of Neurology, Radboud University Medical Center, and Donders Institute for Brain, Cognition and Behavior, P.O. Box 9101, 6500 HB Nijmegen, The Netherlands; 2 Laboratory Medicine, Radboud University Medical Center, and Donders Institute for Brain, Cognition and Behavior, P.O. Box 9101, 6500 HB Nijmegen, The Netherlands; 3 Parkinson Center Nijmegen, Radboud University Medical Center, and Donders Institute for Brain, Cognition and Behavior, P.O. Box 9101, 6500 HB Nijmegen, The Netherlands

**Keywords:** Parkinson’s disease, Multiple system atrophy, Biomarkers, Inflammation

## Abstract

**Background:**

Parkinson’s disease (PD) and atypical parkinsonisms (APD) have overlapping symptoms challenging an early diagnosis. Diagnostic accuracy is important because PD and APD have different prognosis and response to treatment. We aimed to identify diagnostic inflammatory biomarkers of PD and APD in cerebrospinal fluid (CSF) using the multiplex proximity extension assay (PEA) technology and to study possible correlations of biomarkers with disease progression.

**Methods:**

CSF from a longitudinal cohort study consisting of PD and APD patients (PD, *n* = 44; multiple system atrophy (MSA), *n* = 14; vascular parkinsonism (VaP), *n* = 9; and PD with VaP, *n* = 7) and controls (*n* = 25) were analyzed.

**Results:**

Concentrations of CCL28 were elevated in PD compared to controls (*p* = 0.0001). Five other biomarkers differentiated both MSA and PD from controls (*p* < 0.05) and 10 biomarkers differentiated MSA from controls, of which two proteins, i.e. beta nerve growth factor (β-NGF) and Delta and Notch like epidermal growth factor-related receptor (DNER), were also present at lower levels in MSA compared to PD (both *p* = 0.032). Two biomarkers (MCP-1 and MMP-10) positively correlated with PD progression (rho > 0.650; *p* < 0.01).

**Conclusions:**

PEA technique identified potential new CSF biomarkers to help to predict the prognosis of PD. Also, we identified new candidate biomarkers to distinguish MSA from PD.

## Background

Parkinson’s disease (PD) is the most common neurodegenerative disorder of movement affecting 1% of the population worldwide older than 65 years [1]. PD is characterized by motor symptoms: bradykinesia with rigidity and/or rest tremor. The prodromal phase of PD features non-motor symptoms such as olfactory dysfunction, psychiatric symptoms, REM-sleep behavior disorder, autonomic dysfunction, pain and fatigue [1]. At later stages, dementia might also occur. A progressive degeneration of dopaminergic neurons in the substantia nigra pars compacta causes many of the clinical symptoms. Degeneration is associated with the presence of Lewy Bodies, which contain aggregates of the protein α-synuclein. Diagnosis of PD is based on the typical motor symptoms that, however, only appear after 50–80% of dopaminergic neurons have died [2, 3]. Establishing a correct diagnosis of PD can be challenging since its phenotype shares many clinical features with atypical parkinsonisms (APD), especially early in the disease process. APD include multiple system atrophy (MSA), vascular parkinsonism (VaP), dementia with Lewy Bodies (DLB), corticobasal syndrome (CBS) and progressive supranuclear palsy (PSP). Error rates in clinicopathological series of PD and APD patients have been as high as 24%, even when diagnosis was made by movement-disorder specialists [4]. APD has many overlapping symptoms with PD. However, patients with APD generally have an inefficient or transient response to levodopa (current treatment of PD) and a much faster disease progression [5]. Making that distinction based on clinical grounds alone remains difficult, particularly in the first years after the initial manifestation of symptoms. Thus, there is a clear need to discover specific biomarkers that can help clinicians to establish a more timely and accurate differential diagnosis amongst patients suspected of a form of parkinsonism. There is also a need to discover biomarkers that can help clinicians to predict disease progression.

Because of its close connection with the brain, the cerebrospinal fluid (CSF) contains potential biomarker candidates for differential diagnosis. Although the exact etiology of PD and the different forms of APD remain unknown, systemic and cerebral inflammatory processes may be involved in the disease process [6–8]. Microglia, the major type of immune cells of the brain, are activated in PD brains and secrete pro-inflammatory proteins, reactive oxygen species and reactive nitrogen species that contribute to neuronal degeneration [7–10]. Therefore, in the present study, we aimed to discover inflammatory protein biomarkers in CSF that may aid in the differential diagnosis of PD versus APD using a proximity extension assay (PEA). We also aimed to define inflammatory biomarkers that may be related to disease progression.

Recently, it was established that Delta and Notch-like epidermal growth factor-related receptor (DNER), vascular endothelial growth factor A (VEGF-A) and fibroblast growth factor 19 (FGF-19) were differentially expressed in MSA versus PD by using PEA [11]. Since many biomarker discovery studies often lack independent confirmation, we also aimed to confirm these findings in an independent clinical cohort. For this, we used a unique cohort of patients that presented uncertain clinical diagnosis at inclusion and that were followed-up for 12 years.

## Methods

### Patients

A total of 74 cases were selected based on CSF availability from a prospective cohort study performed at the Radboud University Medical Center (Nijmegen, the Netherlands) (*see* Additional file 1) [12]. In this previous study, 156 patients, referred to our center between January 2003 to December 2006 because of parkinsonism and diagnostic uncertainty, were included. Exclusion criteria were age younger than 18 years, history of brain surgery or neurodegenerative disease other than parkinsonism or unstable comorbidity. All patients underwent a structured interview, detailed and standardized neurologic examination, blood collection, lumbar puncture and other ancillary investigations within the 6 following weeks after inclusion. The study design, methods and patient population have been extensively described elsewhere [12]. These patients were followed up for 3 and 10 years and a clinical diagnosis was established by two expert neurologists in movement disorders based on a repeated structured interview and extensive neurological examination. In 2018, 12 years after inclusion, all diagnoses were re-evaluated and updated according to the most recent clinical criteria [12–17]*,* disease course based on the patients’ medical files, follow-up visits and neuropathological examination whenever available. Disease severity and cognitive function were evaluated using the Hoehn and Yahr (HY) scores, the Unified Parkinson’s Disease Rating Scale (UPDRS), the International Cooperative Ataxia Rating Scale (ICARS) and the Mini-Mental State Examination (MMSE). Disease progression was assessed by subtracting the score at follow-up from the score at baseline and dividing by years of follow-up (∆t = 3 years) (Table 1). Neurofilament (NFL) and DJ-1 levels in CSF were obtained from previous biomarker discovery studies using the same cohort [18–20].

**Table 1:** Characteristics of the patients included in the analysis

	Controls	MSA			PD	VaP	PD/VaPD	p value^a^
N	25	MSA-P	MSA-C	MSA-P/C	44	9	7	
		11	2	1				
		14						
Age (at inclusion)	64.5 ± 10.3	61.1 ± 8.0			57.9 ± 9.9	69.5 ± 9.0	70.2 ± 5.1	0.003
Sex (male/female)	11/14	9/5			28/16	7/2	6/1	0.004
Disease duration since first symptoms (months)	N.A.	38.9 ± 38.3			42.0 ± 34.3	25.5 ± 16.11	41.0 ± 19.8	0.556
Disease Severity (baseline)								
HY score	N.A.	2.7 ± 0.8 (14)			2.0 ± 0.6 (43)	2.9 ± 0.7 (9)	2.6 ± 0.9 (7)	0.001
UPDRS-III score	N.A.	30.3 ± 10.0 (14)			28.2 ± 13.8 (42)	33.1 ± 11.3 (9)	40.1 ± 16.8 (7)	0.203
ICARS score	N.A.	10.9 ± 11.5 (11)			2.8 ± 3.4 (39)	10.7 ± 5.1 (7)	8.8 ± 6.1 (6)	0.000
MMSE score	N.A.	27.9 ± 2.7 (13)			28.2 ± 2.1 (44)	26.7 ± 2.9 (8)	26.4 ± 1.5 (7)	0.030
Disease Severity (3 years follow-up)								
HY score	N.A.	4.0 ± 1.1 (10)			2.3 ± 0.8 (41)	4.2 ± 1.0 (6)	2.8 ± 0.9 (5)	0.000
UPDRS-III score	N.A.	33.8 ± 6.8 (5)			29.9 ± 15.1 (39)	44.7 ± 9.3 (4)	39.2 ± 20.6 (5)	0.109
ICARS score	N.A.	16.0 ± 17.9 (5)			3.5 ± 3.2 (35)	20.0 ± 5.7 (4)	5.0 ± 2.5 (5)	0.001
MMSE score	N.A.	27.4 ± 1.1 (5)			27.9 ± 2.9 (34)	27.0 ± 4.1 (4)	24.8 ± 5.7 (5)	0.189
Survival after 12 years (dead/alive)	N.A.	13/1			11/33	9/0	4/3	

Data are represented as mean ± SD (N). p value was considered significant when < 0.05, *MSA* multiple system atrophy, *PD* Parkinson’s disease, *VaP* vascular parkinsonism, *PD/VaP* PD with overlapping VaP, *MSA-P* multiple system atrophy parkinsonian type, *MSA-C* multiple system atrophy cerebellar type, *MSA-P/C* multiple system atrophy mixed parkinsonian and cerebellar, *N.A* not applicable, *HY* Hoehn and Yahr, *UPDRS-III* Unified Parkinson’s Disease Rating Scale part III (motor score), *ICARS* International Cooperative Ataxia Rating Scale, *MMSE* Mini-Mental State Examination. ^a^Kruskal-Wallis test with Bonferroni correction and Chi-square for sex differences

The control group consisted of 25 patients aged above 40 years with neither a neurological nor an inflammatory disease and who underwent a lumbar puncture because of a suspected neurological disorder that was ruled out after extensive investigation.

All participants provided written informed consent and the study was approved by the local Medical Ethics Committee. Usage of CSF leftovers from patients as controls in research projects was approved by the local Medical Ethics Committee.

### Cerebrospinal fluid samples

Lumbar puncture was performed as described previously [12]. CSF samples had no blood contamination (leukocyte number count fewer than 5 cells/μL and erythrocyte number fewer than 200 cells/μL) [21, 22].

### Proximity extension assay (PEA)

Multiplex PEA was conducted using the Proseek Multiplex Inflammation I panel (Olink Bioscience, Uppsala, Sweden). The Proseek kit targeted 92 biomarkers (*see* Additional file 2). Data are expressed as normalized protein expression (NPX) values. NPX is an arbitrary unit on a Log2 scale to normalize data to minimize both intra-assay and inter-assay variation. A high NPX value corresponds to a high protein concentration.

### Data analysis

Statistical analyzes were performed using IBM SPSS Statistics (v.25.0.0.1). Kruskal-Wallis test with Bonferroni correction was performed to assess differences between groups for age, baseline and follow-up parameters as well as disease progression. In general, the Bonferroni correction divides the desired alpha-value by the number of comparisons and uses this number to determine significance. However, the SPSS package uses a mathematical equivalent adjustment; it takes the observed (uncorrected) *p*-value and multiplies it by the number of comparisons made. This corrected p-value is used to conclude significance. If the value is less than 0.05, one can conclude that the difference is significant (https://www.ibm.com/support/pages/calculation-bonferroni-adjusted-p-values). Chi-square test was used to assess sex differences. A p-value < 0.05 was defined as significant.

All proteins of the inflammation panel with more than 35% of missing values (below limit of detection) in the whole cohort were excluded from the analysis (*n* = 39 out of a total of 92) (*See* Additional file 2). Group comparison of NPX values of PEA markers was performed by rank analysis of covariance to correct for age and sex. Briefly, the dependent variables and the covariates were ranked. Then, a linear regression of the ranks of the dependent variable on the ranks of the covariates was performed and the unstandardized residuals were saved. Finally, an ANOVA with Games Howell correction was performed using the unstandardized residuals. The Games Howell post hoc test is used to compare groups with unequal variances. The test was designed based on Welch’s degrees of freedom correction and uses Tukey’s studentized range distribution. Disease progression was calculated using annual change in HY, UPDRS-III, ICARS MMSE and tandem gate scores using the 3 years follow-up and baseline scores. Spearman’s test with 100 bootstrapping was used to correlate the levels of biomarkers at baseline with these annual progression scores. In all cases, a *p* value < 0.05 was considered as statistically significant. Because of the low population power, results of the groups VaP and VaP/PD are not included in the present article.

GraphPad Prism (v.5.00) was used to perform receiver operating characteristic (ROC) curve analysis of biomarkers for PD versus MSA. Biomarkers were combined performing a binary logistic regression and probability values of the logistic regression were used to run the ROC curve analysis.

## Results

In the present study, we analyzed CSF from 44 PD, 14 MSA, 9 VaP, 7 PD with overlapping vascular disease (PD/VaP) and 25 controls. Group comparison revealed that CCL28 was detected at significantly different levels only in PD compared to controls. CCL28 was also the only protein expressed at lower levels in controls than in PD and MSA (*p* = 0.0001; Table 2). Five proteins differentiated both MSA and PD from controls (*p* < 0.05; Table 2). Ten proteins were uniquely differential in MSA compared to controls, of which two proteins, i.e. beta nerve growth factor (β-NGF) and DNER, were also present at lower levels in MSA compared to PD (both *p* = 0.03; Table 2).

**Table 2:** Disease-specific summary of significant different biomarkers in cerebrospinal fluid

Protein	Controls	PD	MSA
CCL28	0.6 ± 0.2^b^	0.9 ± 0.2^a^	0.8 ± 0.2
IL-8	8.4 ± 1.3^bc^	7.6 ± 0.5^a^	7.5 ± 0.4^a^
FGF-19	4.7 ± 0.8^c^	4.2 ± 0.6	3.9 ± 0.7^a^
CD40	8.1 ± 0.5^c^	7.6 ± 0.4	7.5 ± 0.4^a^
PD-L1	4.1 ± 0.6^c^	3.7 ± 0.5	3.5 ± 0.4^a^
TGF-α	6.0 ± 0.6^c^	5.7 ± 0.4	5.5 ± 0.4^a^
SCF	5.2 ± 0.7^c^	4.8 ± 0.5	4.7 ± 0.3^a^
CSF-1	6.9 ± 0.5^c^	6.5 ± 0.4	6.3 ± 0.2^a^
uPA	6.0 ± 0.6^c^	7.0 ± 0.5	6.8 ± 0.4^a^
VEGF-A	9.6 ± 0.7^c^	9.1 ± 0.6	8.9 ± 0.4^a^
CCL23	3.1 ± 1.0^bc^	2.5 ± 0.5^a^	2.5 ± 0.3^a^
CX3CL1	3.2 ± 0.6^bc^	2.6 ± 0.5^a^	2.4 ± 0.3^a^
MCP-2	5.0 ± 0.9^bc^	4.2 ± 0.8^a^	4.2 ± 0.5^a^
CXCL1	6.0 ± 1.2^bc^	4.9 ± 0.5^a^	4.9 ± 0.6^a^
DNER	10.0 ± 0.2^c^	9.9 ± 0.2^c^	9.8 ± 0.1^ab^
β-NGF	1.8 ± 0.5^c^	1.5 ± 0.3^c^	1.3 ± 0.2^ab^

Data are expressed as normalized protein expression (NPX) values (mean ± standard deviation). Data were analyzed using rank analysis of covariance followed by ANOVA with Games Howell as a post hoc test. Only statistically significant (*p* < 0.05) differences are noted. ^a^versus controls; ^b^versus Parkinson’s disease (PD); ^c^versus multiple system atrophy (MSA)

**Fig. 1:**
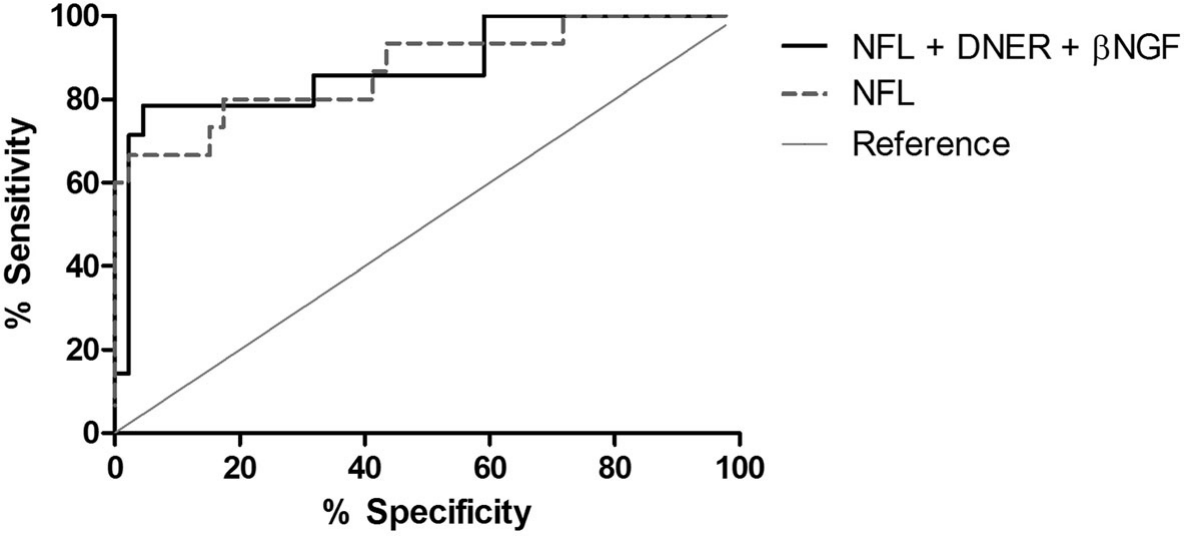
Receiver operating characteristic (ROC) curve analysis of PD versus MSA. The combination of NFL levels with DNER and β-NGF in cerebrospinal fluid (solid black) does not yield better diagnosis accuracy than NFL alone (dashed dark grey) (AUC = 0.88 and 0.87 respectively, *p* value < 0.0001). Reference line in solid clear grey

We assessed the diagnostic value of β-NGF and DNER as potential biomarkers to differentiate MSA from PD using ROC curve analysis. The area under the curve (AUC) for β-NGF was 0.70 (*p* = 0.018) and for DNER 0.71 (*p* = 0.015), whereas the combination of β-NGF and DNER did not yield a better discrimination (AUC = 0.70; *p* = 0.021). We also studied the ability of β-NGF and DNER to distinguish MSA from PD in comparison to other accepted biomarkers (NFL and DJ-1), from which we had available data [19, 20]. NFL (AUC = 0.87; *p* < 0.0001) but not DJ-1 (AUC = 0.72; *p* = 0.038) showed better discrimination power than β-NGF and DNER. The combination of NFL with DNER and β-NGF did not yield an improvement to discriminate PD from MSA (AUC = 0.88; *p* < 0.0001) compared to NFL alone (Fig. 1).

**Fig. 2:**
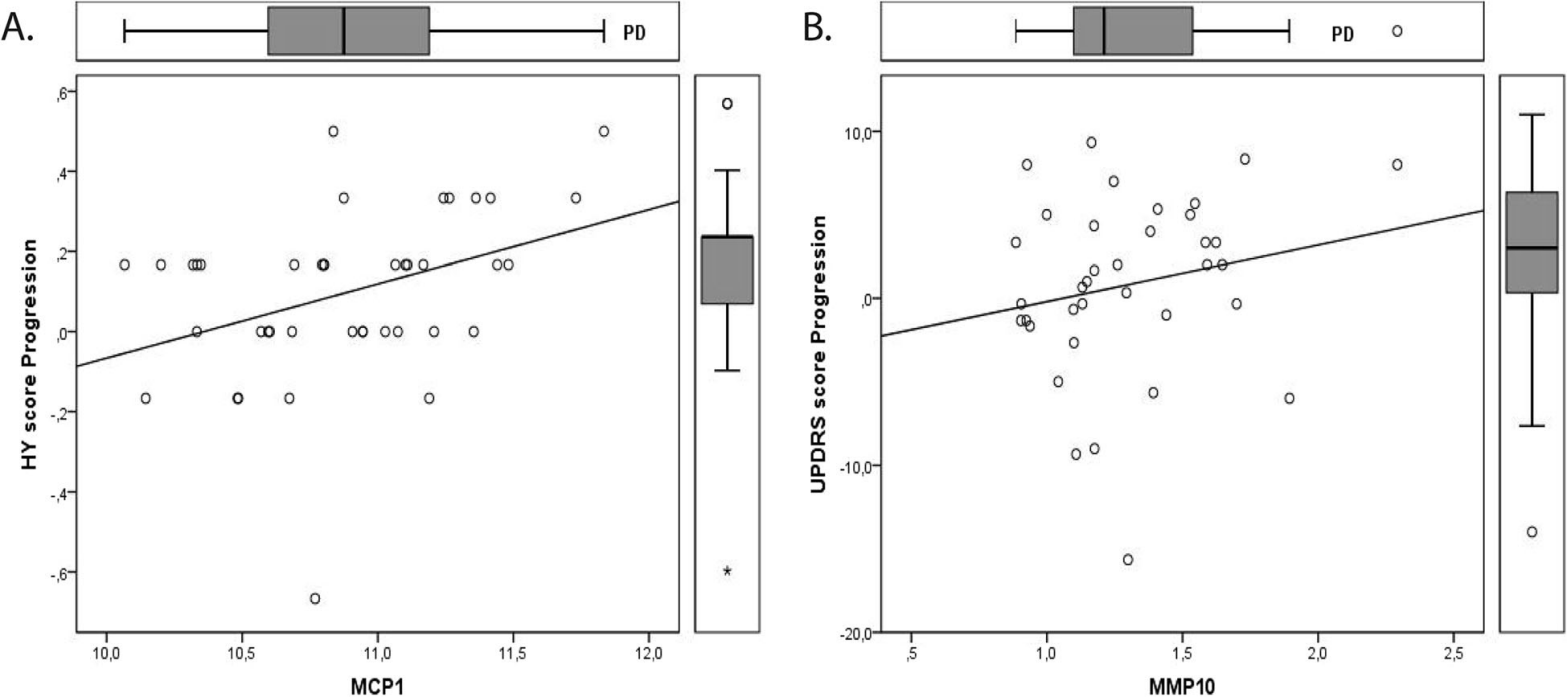
Correlation of biomarkers with Parkinson’s disease (PD) progression. **a.** Correlation between MCP-1 and Hoehn and Yahr (HY) progression score; **b.** Correlation between MMP-10 and unified Parkinson’s Disease rating scale (UPDRS) progression score. Data were analyzed using Spearman correlation. Biomarker values are expressed as normalized protein expression. Right whisker plots represent median, interquartile range, minimum, maximum and outliers of disease score progression. Upper whisker plots represent median, interquartile range, minimum, maximum and outliers of the protein marker levels in cerebrospinal fluid of PD patients. Rho was > 0.600 and p value < 0.01 for both correlations

We also studied the correlation between the inflammatory proteins of the PEA panel and disease progression. These analyses showed that two biomarkers specifically correlated with disease progression in the PD group, i.e. monocyte chemoattractant protein 1 (MCP-1) positively correlated with HY progression (rho = 0.690, *p* = 0.003) and matrix metalloproteinase 10 (MMP-10) positively correlated with the UPDRS progression (rho = 0.651, *p* = 0.006) (Fig. 2). Because of low power, correlations of biomarkers with disease progression in the MSA group are not shown.

## Discussion

Neuroinflammatory mechanisms contribute to the pathology of both PD and APD, which comprise microglia activation, astrocytosis and lymphocyte infiltration as observed in post mortem obtained brain tissue [10]. Moreover, polymorphisms in genes associated with inflammation, such as LRRK2, S100B and NURR1, increase the risk for PD [23–26]. Consequently, the expression levels of inflammatory proteins in CSF might translate into biomarkers for diagnosis and/or prognosis of PD and APD.

From a panel of 92 inflammatory proteins, we identified CCL28 as a biomarker of neuroinflammation that differentiated only PD from controls, which showed a different expression trend (lower expression levels in controls). We also identified 8 biomarkers that differentiated only MSA from controls and 5 biomarkers that differentiated both PD and MSA from controls. Interestingly, DNER and β-NGF could significantly differentiate MSA from PD and controls.

CCL28 (Mucosae-associated epithelial chemokine; MEC) is a chemokine constitutively expressed in mucosal tissue and moderately expressed in small intestine, kidney and brain –in neurons rather than glia cells. CCL28 bridges the innate and adaptative immune response; the C-terminus has antimicrobial activity and the N-terminus mediates lymphocyte migration. Signaling of CCL28 via CCR10 drives the homing of T and B lymphocytes, and via CCR3 the migration of eosinophils [27]. CCL28 was the only biomarker that was up-regulated in PD patients compared to controls. In a mouse model of epilepsy, down-regulated expression of CCL28 in brain tissue was associated with neuronal loss [28]. Another study performed in human PD brain tissue detected lower levels in PD patients compared to controls [29]. This apparent inconsistency with our results might be because they analyzed brain tissue and thus, neuronal expression of CCL28, while we measured levels in CSF detecting expression of other cell types and also systemic inflammation. The elevated levels in CSF might be in line with the idea that viral and microbial infections, as well as altered gut-microbiota, increase the risk of PD or they may even be an early trigger of the disease [30–32]. Another possible reason of elevated levels of CCL28 in CSF might be its release from degenerating neurons.

DNER was observed at higher levels in the CSF of PD patients as compared to MSA patients. DNER is highly expressed in the substantia nigra and, just as Parkin protein, is an activator of the NOTCH1 pathway, which has a role in neuronal and glial cell differentiation and neuroprotection [33, 34]. A down-regulation of DNER protein in MSA might highlight the loss of neuroprotection and thus, the higher disease severity. Interestingly, DNER was also down-regulated in MSA compared to PD to a similar degree as it was described in a previous publication that used the same PEA inflammatory panel for the discovery of biomarkers for PD and APD in two independent cohorts [11].

β-NGF was also significantly down-regulated in MSA compared to PD patients. β-NGF is a trophic factor for sympathetic and sensory fibers found in the peripheral nervous system and in the central nervous system in cholinergic neurons projecting to the cerebral cortex and hippocampus. β-NGF has neuroprotective effects in cholinergic neurons [35–37]. Therefore, the reduction in β-NGF levels may indicate a more advanced neuronal cell loss in MSA. β-NGF was not identified as a potential biomarker to differentiate PD from MSA in the above-mentioned double cohort study [11], which highlights the importance of independent biomarker discovery studies in different cohorts and laboratories.

In contrast to the previously published study using PEA for biomarker identification of PD and APD [11], we did not find significantly different levels of FGF-5, VEGF-A and FGF-19 in PD versus MSA, despite observing a similar trend, i.e. higher expression levels in PD than in MSA. These differences between our and this previous study could possibly be explained by our relatively small number of MSA patients in comparison to PD patients (3-fold difference). A more likely explanation, however, could be that our cohort had unique characteristics, namely all patients who were included in our study had a clear evidence of a form of parkinsonism, but with uncertainty of the specific diagnosis at baseline. Moreover, the diagnosis of the patients has been re-evaluated after 3 years of follow-up and updated again 12 years after inclusion according to the revised clinical criteria. This is unlike the previous study, in which, despite including a few uncertain cases in one of the two cohorts, most patients had a clear diagnosis at CSF withdrawal. Moreover, they followed their patients for no more than 5 years [11, 38, 39].

ROC analysis showed that the combination of DNER and β-NGF do not yield a higher AUC than each of them individually. However, they could be valuable in a bigger panel including biomarkers of different biological processes, such as NFL. In our cohort of study, NFL alone yielded the same diagnostic accuracy as NFL in combination with DNER and β-NGF. The poor added value of these two inflammatory proteins might be caused by their lower expression levels and the remodulation of the immune system at older age, losing the ability to fine-tune inflammation [40]. Further studies need to determine the positive impact of adding these inflammatory proteins to a larger diagnostic panel to discriminate PD from MSA patients but our data suggests that such impact is likely to be minimal.

It can be hypothesized that PD (or MSA) patients with more pronounced neuroinflammation than others will have a more severe disease progression. For this reason, we correlated levels of CSF proteins at baseline with disease progression over a 3-year time-frame. Both MCP-1 and MMP-10 showed a significant positive correlation with parameters of PD progression. MCP-1 plays an important role in monocyte recruitment and propagation of inflammation. Previous studies showed that plasma levels of MCP-1 correlated with cognitive decline in patients with Alzheimer’s disease [41]. Other studies in mouse models suggest that MCP-1 causes neuronal loss and that its downregulation is neuroprotective [42, 43]. MMP-10 is a secreted metalloproteinase with a key role on modulation of macrophage activation and function. MMP-10 is not expressed in unchallenged tissues, but is increased in response to a variety of insults [44]. Thus, a positive correlation of MMP-10 CSF levels with disease progression might indicate increased inflammation and neuronal loss.

The major strength of our study is the uniqueness of our patient cohort. Patients with diagnostic uncertainty were included in the study and their diagnosis was reevaluated after 3 and 12 years. Thus, our study exactly reflects the clinical situation when biomarkers are actually needed, i.e. biomarkers have diagnostic value when the diagnosis is not yet clear. Unlike our study, many biomarker studies have been performed with patients with a clear-cut diagnosis, but in such situations biomarkers will not add anything to the diagnostic work-up.

Our study also presents four limitations. First, our group of MSA patients was relatively small, which may have affected our analyses. However, the patients were very well-defined as a result of the long-term follow-up. Second, the final diagnosis was based on clinical evaluation according to international diagnostic criteria, but has not been confirmed yet by neuropathologic examination. This may have caused potential misdiagnoses, but we have reduced this risk by the very long follow-up of the patients. For most patients, a ‘silver standard’ diagnosis can be made after some 3 years of follow-up, when the rate of progression is known, new red flags may have appeared, and the levodopa responsiveness has been tested. Third, the disease progression is calculated based on the 3-year follow-up scores. A stronger correlation of biomarkers with prognosis might be observed with data from longer follow-up periods. Fourth, the study did not include patients with other forms of atypical parkinsonism, such as PSP, CBS and DLB, due to the small number of patients with these diagnoses included in the longitudinal study.

## Conclusions

In summary, our results show 16 differentially expressed proteins. Among these proteins, DNER and β-NGF are especially interesting as they can discriminate MSA from PD, although our data show that their contribution may be limited. Our study also suggests that baseline CSF levels of MCP-1 and MMP-10 may serve as biomarkers of PD progression. This finding, however, requires further replication in independent cohorts.

## Supplementary information

**Additional file 1.** Flowchart of follow-up of patients included in this study. Patients with PSP, DLB and CBS were not selected for the study because of low numbers. Patients with another or uncertain diagnosis were also not selected. VaP and VaP/PD patients were included in the statistical analysis but data is not shown because of the low number of patients in these groups. PD: Parkinson’s disease; MSA: multiple system atrophy; PSP: progressive supranuclear palsy; DLB: dementia with Lewy bodies; VaP: vascular parkinsonism; CBS: corticobasal syndrome; PD/VaP: PD with overlapping VaP. In brackets the number of patients per group. The black squares represent the patients the study focused on.

**Additional file 2.** Proteins included in the Olink inflammation panel. In bold are highlighted the proteins that were excluded because their levels in cerebrospinal fluid were lower the limit of detection in more than 35% of the samples.

## Data Availability

The datasets used and analyzed during the current study are available from the corresponding author on reasonable request indefinitely after publication date.

## Abbreviations

APD: Atypical parkinsonisms
AUC: Area under the curve
CBS: Corticobasal syndrome
CCL28: Mucosae-associated epithelial chemokine
CSF: Cerebrospinal fluid
DLB: Dementia with Lewy bodies
DNER: Delta and notch like epidermal growth factor-related receptor
FGF-19: Fibroblast growth factor 19
HY: Hoehn and Yahr scores
ICARS: International cooperative ataxia rating scale
MCP-1: Monocyte chemoattractant protein 1
MMP-10: Matrix metalloproteinase 10
MMSE: Mini-mental state examination
MSA: Multiple system atrophy
NFL: Neurofilament
NPX: Normalized protein expression
PD: Parkinson’s disease
PD/VaP: Parkinson’s disease with overlapping vascular parkinsonism
PEA: Proximity extension assay
PSP: Progressive supranuclear palsy
ROC: Receiver operating characteristic
UPDRS: Unified Parkinson’s disease rating scale
VaP: Vascular parkinsonism
VEGF-A: Vascular endothelial growth factor A
β-NGF: Beta nerve growth factor
