# Supplementary material for: Inflammation biomarker discovery in Parkinson’s disease and atypical parkinsonisms
Source: BMC Neurol. 2020 Jan 17;20:26. doi: 10.1186/s12883-020-1608-8 (PMC6967088; doi:10.1186/s12883-020-1608-8)
Supplement: Supplementary file 1 — Additional file 1. Flowchart of follow-up of patients included in this study. Patients with PSP, DLB and CBS were not selected for the study because of low numbers. Patients with another or uncertain diagnosis were also not selected. VaP and VaP/PD patients were included in the statistical analysis but data is not shown because of the low number of patients in these groups. PD: Parkinson’s disease; MSA: multiple system atrophy; PSP: progressive supranuclear palsy; DLB: dementia with Lewy bodies; VaP: vascular parkinsonism; CBS: corticobasal syndrome; PD/VaP: PD with overlapping VaP. In brackets the number of patients per group. The black squares represent the patients the study focused on. [file 12883_2020_1608_MOESM1_ESM.docx]

Patients selected for the statistical analysis

Patients from a prospective cohort (118)

Patients selected for Olink analysis (97)

Diagnosis at baseline

Diagnosis after 3-years follow-up

Diagnosis after 12-years follow-up

PD (47)

MSA (26)

PSP (3)

DLB (2)

VaP (16)

CBS (3)

PD/VaP (0)

PD (43)

MSA (24)

PSP (3)

DLB (2)

VaP (16)

CBS (3)

PD/VaP (6)

PD (46)

MSA (15)

PSP (1)

DLB (2)

VaP (9)

CBS (0)

PD/VaP (7)

Other (5)

Uncertain (12)

PD (46)

MSA (15)

VaP (9)

PD/VaP (7)
