# Supplementary material for: Inflammation biomarker discovery in Parkinson’s disease and atypical parkinsonisms
Source: BMC Neurol. 2020 Jan 17;20:26. doi: 10.1186/s12883-020-1608-8 (PMC6967088; doi:10.1186/s12883-020-1608-8)
Supplement: Supplementary file 2 — Additional file 2. Proteins included in the Olink inflammation panel. In bold are highlighted the proteins that were excluded because their levels in cerebrospinal fluid were lower the limit of detection in more than 35% of the samples. [file 12883_2020_1608_MOESM2_ESM.docx]

| Biomarkers in Olink inflammation panel | |
| --- | --- |
| Adenosine Deaminase | ADA |
| Artemin | **ARTN** |
| Axin-1 | **AXIN1** |
| Beta-nerve growth factor | Beta-NGF |
| C-C motif chemokine 19 | CCL19 |
| C-C motif chemokine 20 | **CCL20** |
| C-C motif chemokine 23 | CCL23 |
| C-C motif chemokine 25 | CCL25 |
| C-C motif chemokine 28 | CCL28 |
| C-C motif chemokine 3 | CCL3/MIP-1 alpha |
| C-C motif chemokine 4 | CCL4 |
| C-X-C motif chemokine 1 | CXCL1 |
| C-X-C motif chemokine 10 | CXCL10 |
| C-X-C motif chemokine 11 | CXCL11 |
| C-X-C motif chemokine 5 | CXCL5 |
| C-X-C motif chemokine 6 | CXCL6 |
| C-X-C motif chemokine 9 | CXCL9 |
| Caspase-8 | **CASP-8** |
| CD40L receptor | CD40 |
| *CUB domain-containing protein 1* | CDCP1 |
| Cystatin D | CST5 |
| Delta and Notch-like epidermal growth factor-related receptor | DNER |
| Eotaxin | CCL11 |
| Eukaryotic translation initiation factor 4E-binding protein 1 | 4E-BP1 |
| Fibroblast growth factor 19 | FGF-19 |
| Fibroblast growth factor 21 | **FGF-21** |
| Fibroblast growth factor 23 | **FGF-23** |
| Fibroblast growth factor 5 | FGF-5 |
| Fms-related tyrosine kinase 3 ligand | Flt3L |
| Fractalkine | CX3CL1 |
| Glial cell line-derived neurotrophic factor | **GDNF** |
| Hepatocyte growth factor | HGF |
| Interferon gamma | **IFN-gamma** |
| Interleukin-1 alpha | **IL-1 alpha** |
| Interleukin-10 | **IL-10** |
| Interleukin-10 receptor subunit alpha | IL-10RA |
| Interleukin-12 subunit beta | IL-12B |
| Interleukin-13 | **IL-13** |
| Interleukin-15 receptor subunit alpha | **IL-15RA** |
| Interleukin-17A | **IL-17A** |
| Interleukin-17C | **IL-17C** |
| Interleukin-18 | IL-18 |
| Interluekin-18 receptor 1 | IL-18R1 |
| Interleukin-2 | **IL-2** |
| Interleukin-2 receptor subunit beta | **IL-20** |
| Interleukin-20 | **IL-20RA** |
| Interleukin-20 receptor subunit alpha | **IL-20RA** |
| Interleukin-22 receptor subunit alpha-1 | **IL-22RA1** |
| Interleukin-24 | **IL-24** |
| Interleukin-33 | **IL-33** |
| Interleukin-4 | **IL-4** |
| Interleukin-5 | **IL-5** |
| Interleukin-6 | IL-6 |
| Interleukin-7 | IL-7 |
| Interleukin-8 | IL-8 |
| Latency-associated peptide transforming growth factor beta-1 | LAP TGF-beta-1 |
| Leukemia inhibitory factor | **LIF** |
| Leukemia inhibitory factor receptor | LIF-R |
| Macrophage colony-stimulating factor 1 | CSF-1 |
| Matrix metalloproteinase-1 | MMP-1 |
| Matrix metalloproteinase-10 | MMP-10 |
| Monocyte chemotactic protein 1 | MCP-1 |
| Monocyte chemotactic protein 2 | MCP-2 |
| Monocyte chemotactic protein 3 | **MCP-3** |
| Monocyte chemotactic protein 4 | **MCP-4** |
| Natural killer cell receptor 2B4 | CD244 |
| Neurotrophin-3 | **NT-3** |
| Neurturin | **NRTN** |
| Oncostatin-M | **OSM** |
| Osteoprotegerin | OPG |
| Programmed cell death 1 ligand 1 | PD-L1 |
| Protein S 100-A12 | **EN-RAGE** |
| Signaling lymphocytic activation molecule | **SLAMF1** |
| SIR2-like protein 2 | SIRT2 |
| STAM-binding protein | **STAMBP** |
| Stem cell factor | SCF |
| Sulfotransferase 1A1 | **STA1A1** |
| T cell surface glycoprotein CD6 isoform | **CD6** |
| T-cell surface glycoprotein | CD5 |
| T-cell surface glycoprotein CD8 alpha chain | **CD8A** |
| Thymic stromal lymphopoietin | **TSLP** |
| TNF-beta | **TNFB** |
| TNF-related activation-induced cytokine | **TRANCE** |
| TNF-related apoptosis-inducing ligand | TRAIL |
| Transforming growth factor alpha | TGF-alpha |
| Tumor necrosis factor (Ligand) superfamily member 12 | TWEAK |
| Tumor necrosis factor | **TNF** |
| Tumor necrosis factor ligand superfamily member 14 | TNFSF14 |
| Tumor necrosis factor receptor superfamily member 9 | TNFRSF9 |
| Urokinase-type plasminogen activator | uPA |
| *Vascular endothelial growth factor A* | VEGF-A |
